# Iron Molybdate Fe_2_(MoO_4_)_3_ Nanoparticles: Efficient Sorbent for Methylene Blue Dye Removal from Aqueous Solutions

**DOI:** 10.3390/molecules25215100

**Published:** 2020-11-03

**Authors:** Ahmed Mohmoud, Souad Rakass, Hicham Oudghiri Hassani, Fethi Kooli, Mostafa Abboudi, Sami Ben Aoun

**Affiliations:** 1 Petroleum Technology, Operated Offshore Oil Field Development, Qatar Petroleum, Doha P.O. Box 3212, Qatar; caadil77@yahoo.co.uk; 2 Department of Chemistry, Faculty of Science, Taibah University, Al-Madinah Al-Munawarah P.O. Box 30002, Saudi Arabia; abboudi14@hotmail.com (M.A.); sbenaoun@hotmail.com (S.B.A.); 3 Laboratory of Applied Organic Chemistry (LCOA), Chemistry Department, Faculty of Sciences and Techniques, Sidi Mohamed Ben Abdellah University, P.O. Box 2202, Imouzzer Road, 30000 Fez, Morocco; 4 Engineering Laboratory of Organometallic, Molecular Materials and Environment (LIMOME), Faculty of Sciences, Chemistry Department, Sidi Mohamed Ben Abdellah University, P.O. Box 1796 (Atlas), 30000 Fez, Morocco; oudghiri_hassani_hicham@yahoo.com; 5 Department of Chemistry, Faculty of Science, Islamic University of Madinah, Al-Madinah Al-Munawarah 42351, Saudi Arabia; fkooli@yahoo.com

**Keywords:** nanosorbent, Fe_2_(MoO_4_)_3_, removal, methylene blue, regeneration

## Abstract

The present study investigated iron molybdate (Fe_2_(MoO_4_)_3_), synthesized via a simple method, as a nanosorbent for methylene blue (MB) dye removal from aqueous solutions. Investigations of the effects of several parameters like contact time, adsorbent dose, initial dye concentration, temperature and pH were carried out. The results showed that MB removal was affected, significantly, by adsorbent dose and pH. Interestingly, lower values of adsorbent dose resulted in the removal of higher amounts of MB. At the optimum pH, the removal efficiency of 99% was gained with an initial MB concentration of ≤60 ppm. The kinetic study specified an excellent correlation of the experimental results with the pseudo-second-order kinetics model. Thermodynamic studies proved a spontaneous, favorable and endothermic removal. The maximum amount of removal capacity of MB dye was 6173 mg/g, which was determined from the Langmuir model. The removal efficiency was shown to be retained after three cycles of reuse, as proven by thermal regeneration tests. The presence and adsorption of the dye onto the Fe_2_(MoO_4_)_3_ nanoparticle surface, as well as the regeneration of the latter, was ascertained by scanning electron microscopy (SEM) and Fourier-transform infrared spectroscopy (FTIR). These findings are indicative that the investigated nanosorbent is an excellent candidate for the removal of MB in wastewater.

## 1. Introduction

The textile and dyeing industries are major problem sources of water pollution [1,2]. Dye effluent released into clean water causes a variety of health hazards in marine organisms, freshwater sources and humans [2]. Thus, dyes are considered to be highly toxic and carcinogenic, and their removal is of great interest.

Therefore, several methods have been developed and employed for the removal and/or degradation of hazardous organic dyes from contaminated wastewater, such as photodegradation, extraction, adsorption, membrane separation, coagulation, flocculation, chemical oxidation, ion exchange and biological treatment [3,4,5,6,7,8,9]. Among all the techniques, adsorption by natural and synthetic adsorbents has been widely used because of its simplicity and efficiency for toxic dye removal in wastewater [10,11,12,13,14,15].

Several natural adsorbents are effective in removing dyes from aqueous effluents [10,11,16,17], of which activated carbon is the most commonly used, mainly due to its superior adsorption efficiency [18,19]. Nevertheless, commercially available activated carbon is very expensive [20].

There is therefore a great need to develop efficient adsorbents showing superior performance in their ability to remove pollutants and having suitable properties, such as ease of separation from the solution, regeneration and efficiency, even after several cycles of use, in order to enable the recovery of valuable compounds.

In recent decades, metal oxides as potential materials with different capabilities have been studied [21]. The family of metal molybdates is one of the most promising examples of the mixed metal oxides, which have been extensively studied in recent years [22,23,24,25].

Metal molybdate compounds with the formula MMoO_4_ or M_2_(MoO_4_)_3_ are important inorganic materials that have attracted great research interest, because they have important industrial applications. These include photocatalytic materials, humidity sensors, scintillator materials, photoluminescent compounds and optical fibers [26,27]; microwave applications and electrochemical and magnetic properties are also key features of these compounds. Metal molybdate compounds synthesized in the nanoscale were also used for environmental applications such as the removal of dyes from water by adsorption [23,28], oxidation of methylene blue dye [29] and photocatalytic oxidation of dyes [30].

Among all molybdenum-containing mixed oxides, the most studied is perhaps iron molybdate Fe_2_(MoO_4_)_3_. It has garnered an exponentially increasing degree of interest, it has been synthesized at the nanoscale by different methods and it has been involved in many environmental and industrial applications [31]. In fact, iron molybdate has important applications in solid oxide fuel cells, sodium and lithium ion batteries, catalysis and sensors [32], propylene oxide production via the oxidation of propylene [33,34], methanol oxidation to formaldehyde [35] and photo-combined heterogeneous activation of persulfate for the removal of micropollutants [36]. In addition, the coprecipitation preparation of Fe_2_(MoO_4_)_3_ nanopowder was applied for the photocatalytic degradation of rhodamine B with an efficiency of ~97% [37].

In the present work, iron molybdate nanoparticles prepared at a rather low temperature via a relatively cost-effective and very simple procedure, as previously described in the literature [22], were investigated as an adsorbent for MB dye removal. The effects of different parameters such as solution pH, temperature, contact time, adsorbent dosage and initial dye concentration on the removal of MB by the synthesized Fe_2_(MoO_4_)_3_ nanosorbents were studied. In addition, the adsorption isotherms and kinetics were evaluated. Furthermore, the removal efficiency after regeneration of the used nanosorbent by calcination at high temperature was evaluated.

## 2. Results and Discussion

### 2.1. MB Removal

#### 2.1.1. pH Effect

**Figure 1:**
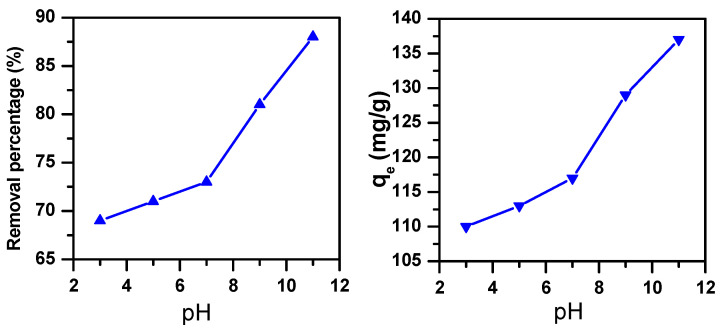
Removal efficiency of Fe_2_(MoO_4_)_3_ in an 80-ppm methylene blue (MB) solution as a function of pH (m_ads_ = 0.05 g, T = 20 °C, t = 30 min).

An essential parameter in terms of controlling the dye removal is pH [38]. While it does not modify the adsorbent site separation, it nevertheless changes the chemistry and the structure of the dye [39]. Therefore, the effect of pH on MB removal using iron molybdate Fe_2_(MoO_4_)_3_ nanosorbent was investigated by varying its values between 3 and 11 at room temperature (i.e., 20 °C) with an initial concentration of 80 ppm. As shown in Figure 1, there is a pronounced effect of pH on MB removal. For instance, an increasing removal percentage from 69% to 88% was observed with increasing pH values from 3 to 11. The same trend was also observed for the dye removal amount per adsorbent unit mass at equilibrium (q_e_), which increased from 110 to 137 mg/g. There was a strong electrostatic interaction of the charges between the MB dyes and the Fe_2_(MoO_4_)_3_ adsorbent, and this was shown by increasing the pH values, which resulted in a higher percentage obtained. In fact, the hydroxyl group (OH^−^) in the solution at pH 11 favors the positive charge of the MB, which has a pKa equal to 3.8 [40]. However, at acidic values, the lower removal efficiency could be linked to the excess of proton ions in the solution competing with the basic dye cations on the removal sites of Fe_2_(MoO_4_)_3_. Similar findings were reported by Kooli et al. [41] in a study of waste bricks applied as a promising removal agent for basic blue 41 from aqueous solutions. Thus, the best value for MB removal using Fe_2_(MoO_4_)_3_ nanosorbent was shown at pH 11.

#### 2.1.2. Adsorbent Dose Effect

**Figure 2:**
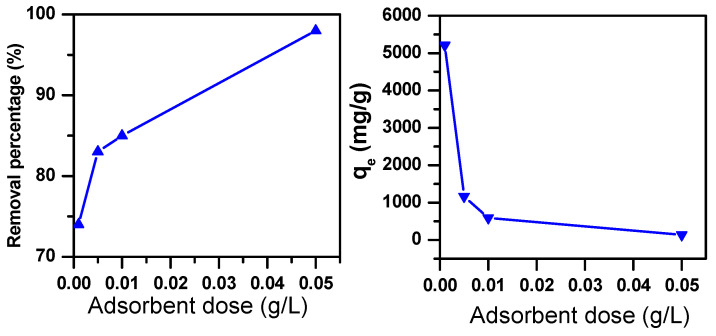
Removal efficiency of Fe_2_(MoO_4_)_3_ in a 70-ppm MB solution as a function of the adsorbent dose (t = 30 min, T = 20 °C).

The adsorbent dose is regarded as one of the important parameters in the adsorption processes [42]. MB dye removal using Fe_2_(MoO_4_)_3_, with an initial dye concentration of 70 ppm, was explored by varying the dose of the adsorbent between 0.001 and 0.05 g/L. As can be depicted from Figure 2, the percentage (%) of removed MB increased, and its concentration (mg/g) decreased when the adsorbent dose increased from 0.001 to 0.05 g/L. This was an expected tendency, since the active sites of the adsorbent’s surface area increase with the increasing adsorbent dose, which therefore leads to an increasing amount of removed MB [43].

#### 2.1.3. Initial Dye Concentration and Contact Time Effect

**Figure 3:**
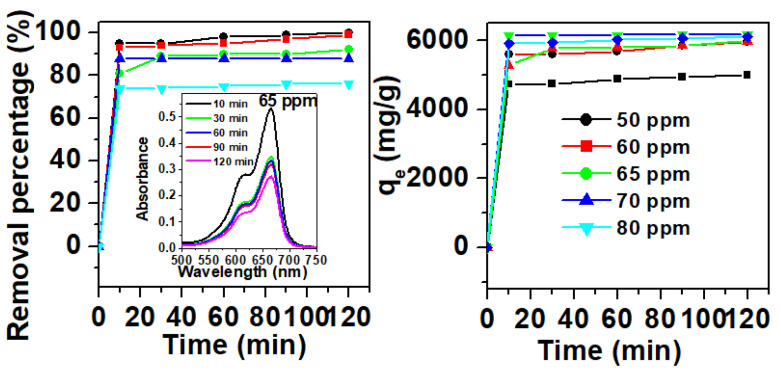
Removal efficiency of Fe_2_(MoO_4_)_3_ for methylene blue (MB) as a function of initial dye concentration and contact time (m_adsorbent_ = 0.001 g, T = 20 °C). Inset: UV spectra of MB solutions (65 ppm) after contact with Fe_2_(MoO_4_)_3_ as a function of time.

Removal studies, in light of the effect of initial MB dye concentration and contact time, were conducted at pH 11, and the results are shown in Figure 3. A percentage of 95% removal was achieved within 30 min for both C_i_ of 50 and 60 ppm, which increased to 99% after a 120-min contact time. As for a C_i_ value of 65 ppm, the removal percentage maximum (92%) was reached with a contact time of 120 min. However, removal maxima of 88% and 76% were obtained with C_i_ values of 70 and 80 ppm, respectively, after a 120-min contact time. Thus, the removal capacity increased notably from 4999 mg/g to 6179 mg/g with increasing initial concentrations of dye from 50 to 80 ppm. Such a trend could be attributed to the initially abundant empty sites onto the Fe_2_(MoO_4_)_3_ surface, which, as a consequence of the sorption process, gradually decreased by filling up these sites with increasing contact times [44].

#### 2.1.4. Temperature Effect

**Figure 4:**
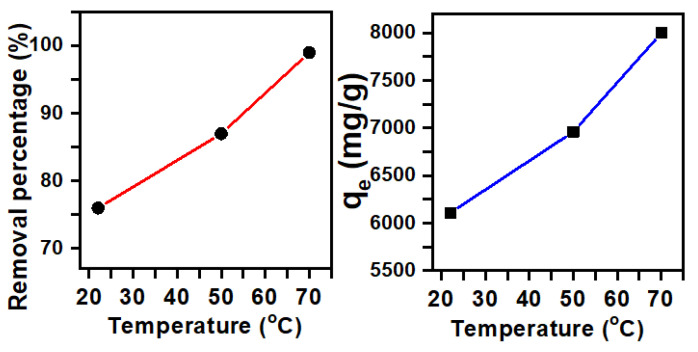
The removal efficiency of Fe_2_(MoO_4_)_3_ in a 80-ppm MB solution as a function of temperature (t = 30 min, pH = 11).

Temperature is another parameter of prime importance that has a great impact on dye removal [45]. The process of removing the MB dye was investigated from 20 to 70 °C, as can be seen in Figure 4. The effect of temperature studies showed that the percentage removal increased from 76% to 99% at an initial dye concentration of 80 ppm, with an increased removal capacity from 6109 mg/g to 7999 mg/g. In fact, the removal motion of the adsorbent sites improved with increasing temperature, which, in turn, caused the motion of the dye molecules to increase [46].

**Figure 5:**
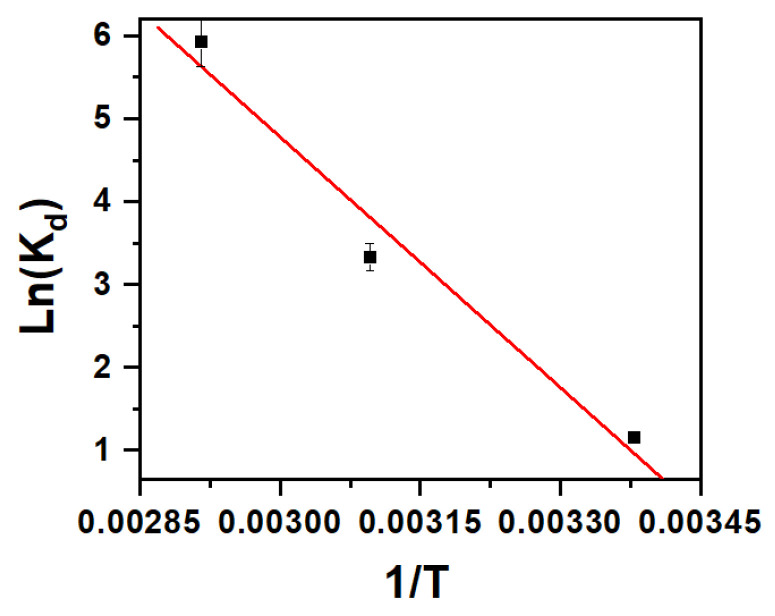
Van ’t Hoff plot showing the effect of temperature on MB removal by Fe_2_(MoO_4_)_3_.

Thermodynamic factors are important parameters in the adsorption processes [47]. The probability and the adsorption mechanism are predictable in the light of thermodynamic parameters [47]. These can be evaluated by means of the following equations:

(1) $$\Delta G^{o}=-{\mathrm{RTLnK}}_{d}$$

(2) $$K_{d}=\frac{C_{a}}{C_{e}}$$

(3) $${\mathrm{LnK}}_{d}=\frac{\Delta S^{o}}{R}-\frac{\Delta H^{o}}{\mathrm{RT}}$$

where K_d_ is the distribution constant, T is the absolute temperature (K), R is the gas constant (J.mol^−1^.K^−1^), ΔG° is the free energy, C_a_ is the amount of dye adsorbed at equilibrium, C_e_ is the equilibrium concentration (mol/L) and ΔH° and ΔS° are the standard enthalpy and standard entropy, respectively. The values of ∆S° and ∆H° were determined from the intercept and slope of the plot ln K_d_ versus 1/T (Figure 5), and ∆G° values were calculated from Equation (1). All data are shown in Table 1.

The negative sign of ∆G° indicated a favorable and spontaneous adsorption. The removal of MB dye, as indicated by the positive value of ∆H° (83.79 KJ.mol^−1^), was proven to occur via a physisorption process [48]. The positive values of ∆S° were proof of the increased randomness and disorder at the solid–solution interface of Fe_2_(MoO_4_)_3_ and MB. The adsorbate molecules caused the adsorbed water molecules to move, and consequently, an additional translational energy was gained, resulting in a random system taking place [49].

### 2.2. Kinetic Study

A kinetic test for the removal of MB was investigated in order to provide an indication regarding the adsorption system [38].

The kinetics of MB dye elimination using Fe_2_(MoO_4_)_3_ nanosorbent were evaluated using intraparticle diffusion and pseudo-first-order and pseudo-second-order kinetic models. The equations of the considered models are given in Table 2.

**Figure 6:**
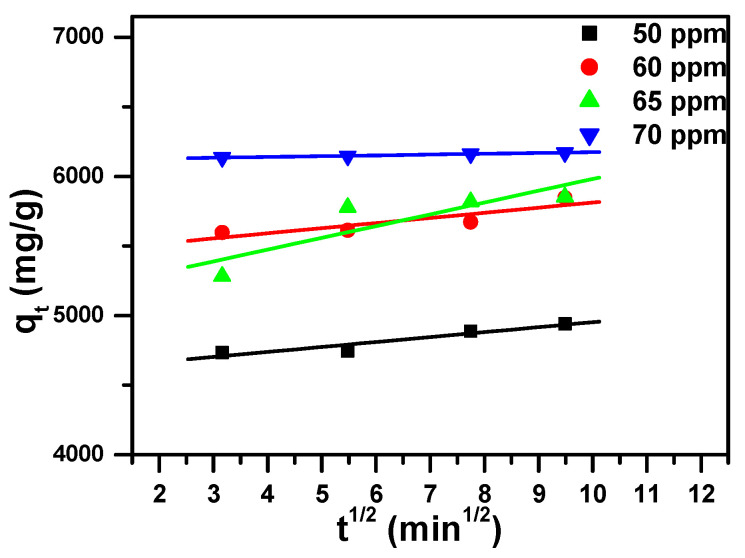
Intraparticle diffusion model plot showing the effect of contact time and initial dye concentration of MB removal by Fe_2_(MoO_4_)_3_.

**Figure 7:**
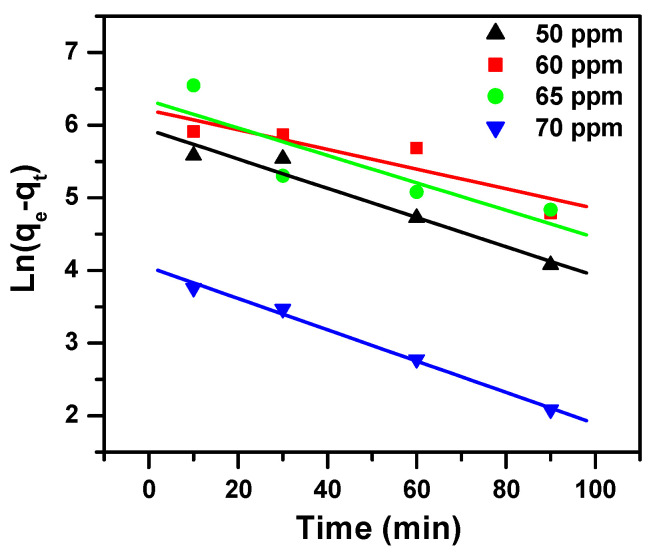
Pseudo-first-order model plot showing the effect of contact time and initial dye concentration on MB removal using Fe_2_(MoO_4_)_3_.

**Figure 8:**
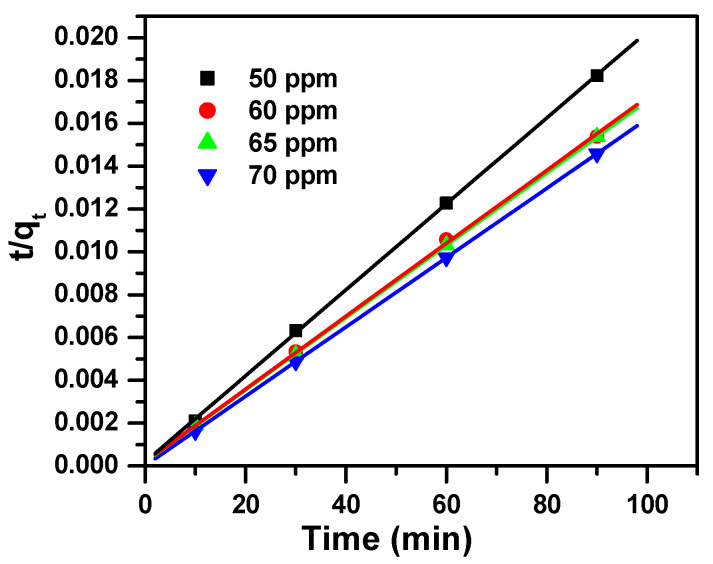
Pseudo-second-order model plot showing the effect of contact time and initial dye concentration of MB removal by Fe_2_(MoO_4_)_3_.

The three model parameters, namely intraparticle diffusion, pseudo-first-order and pseudo-second-order, are arranged in Table 3 and shown in Figure 6, Figure 7 and Figure 8, respectively. These models varied in the values of correlation coefficients (R^2^) of the linear regressions. These values were estimated as follows: 0.748 to 0.993 for intraparticle diffusion, 0.752 to 0.993 for pseudo-first-order model and 0.999 to 1.000 for pseudo-second-order model for the studied concentrations. Since the R^2^ value was equal to or near 1 for the pseudo-second-order model, the latter fit very well to the experimental data.

### 2.3. Adsorption Isotherms

It is essential to examine adsorption isotherms because of the information they provide when planning to use the adsorption method [50]. In the present work, we studied the four major adsorption models, namely Freundlich, Langmuir, Dubinin-Radushkevich and Temkin. These are governed by the equations presented in Table 4.

**Figure 9:**
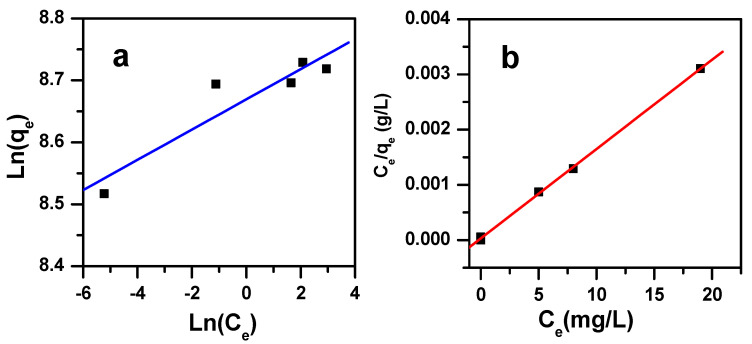
Plots of (**a**) the Freundlich and (**b**) Langmuir isotherms displaying the initial dye concentration effect on the removal of MB by Fe_2_(MoO_4_)_3_.

The Dubinin-Radushkevich, Temkin, Freundlich and Langmuir models were studied and attempted to fit the experimental data. The model parameters and the regression correlation coefficients (R^2^) are given in Table 5, as extracted from Figure 9. The highest value for R^2^ (0.999) was obtained from the Langmuir model, while the fittings of the Freundlich and Temkin models showed the lowest values of R^2^ (0.866 and 0.870, respectively), whereas an intermediary value was achieved for the D-R model (R^2^ = 0.971). Accordingly, the Langmuir isotherm had the best fit with the experimental results, suggesting that the dye removal proceeded via the formation of an MB monolayer onto the Fe_2_(MoO_4_)_3_ adsorbent surface, with a high adsorption capacity of 6173 mg/g, leading to a homogenous surface. On the other hand, the separation factor R_L_, ranging from 0.0024 to 0.0038, indicated a favorable dye removal by Fe_2_(MoO_4_)_3_. Therefore, the investigated nanosorbent had excellent removal efficiency when compared to other materials (Table 6).

### 2.4. Regeneration and Characterization of the Fe_2_(MoO_4_)_3_ Nanosorbent

#### 2.4.1. Regeneration Efficiency

The repeatability and regeneration of the nanosorbent are very important parameters for its eventual practical applications. In the literature, several regeneration procedures were suggested, such as microwave irradiation, bio-regeneration, supercritical regeneration, chemical extraction, thermal treatment, etc. [17,45,57,58,59]. The thermal regeneration used in the present work was similar to that discussed in our previous work [45]. In this research, this thermal treatment method was tested for the regeneration process, as the structure of the Fe_2_(MoO_4_)_3_ removal agent was stable.

The results showed that Fe_2_(MoO_4_)_3_ was prone to regeneration by thermal treatment.

**Figure 10:**
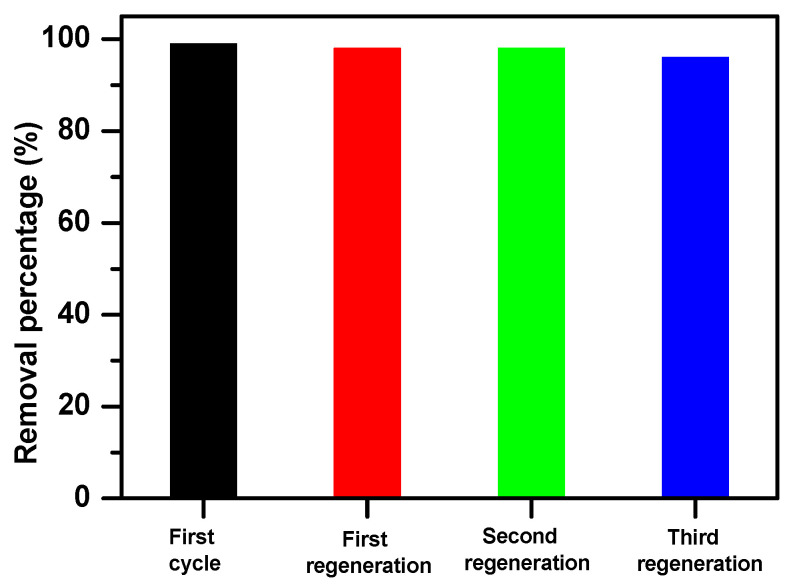
Recycled efficiencies of Fe_2_(MoO_4_)_3_ for the removal of methylene blue (60 ppm, 0.001 g, 30 min).

Figure 10 shows the recycled efficiency of Fe_2_(MoO_4_)_3_ for the removal of methylene blue for three cycles. In fact, the results showed a decrease of dye removal from 99% to 96%, with a decreasing removal capacity from 5932 to 5777 mg/g. The adsorbent regeneration through calcination at 400 °C under air atmosphere was shown to be extremely efficient, in addition to its excellent reusability, as suggested by the observed high removal efficiencies.

#### 2.4.2. Fourier-Transform Infrared Spectroscopy

**Figure 11:**
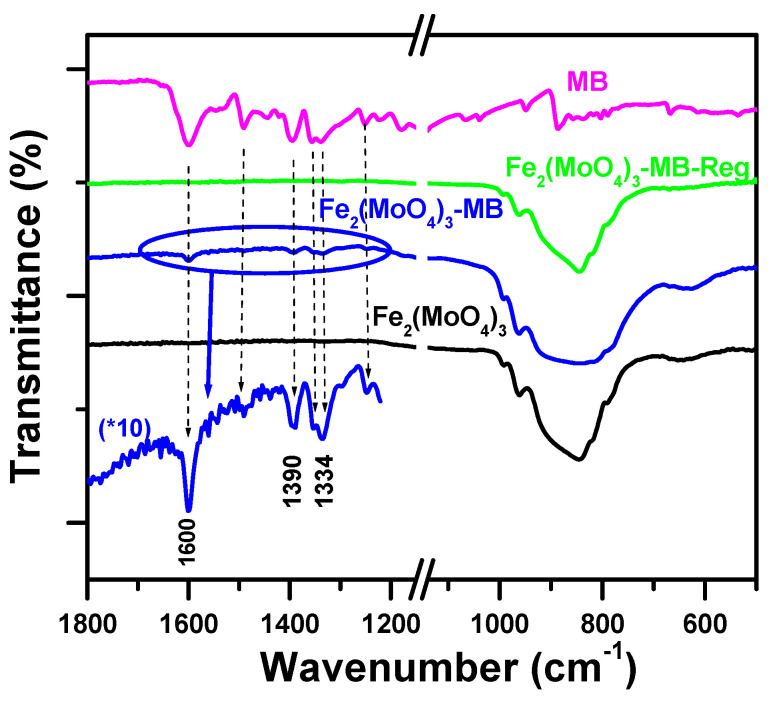
Fourier-transform infrared (FTIR) spectra of Fe_2_(MoO_4_)_3_, Fe_2_(MoO_4_)_3_–MB, Fe_2_(MoO_4_)_3_–MB-Reg and MB.

With the aim of fully elucidating the removal process of MB dye by Fe_2_(MoO_4_)_3_ nanosorbent, an FTIR spectroscopic study was carried out on the material prior to and after exposure to MB dye. Figure 11 displays the spectra of the Fe_2_(MoO_4_)_3_ nanosorbent in both cases. As can be noticed, clear flexing and stretching vibrations characteristic of the metal-oxygen bonds were situated at frequencies between 700 and 1000 cm^−1^, corresponding to the vibrations of the Mo–O bond of the MoO_4_ tetrahedra in the Fe_2_(MoO_4_)_3_ [60]. The pure MB spectrum exhibited bands between 1700 and 1000 cm^−1^ [61], while after MB adsorption, additional bands located at 1600 cm^−1^ were shown in the FTIR spectrum of (Fe_2_(MoO_4_)_3_–MB). These were attributed to MB C=C bond stretching, inferring the presence of MB as a result of its attachment to Fe_2_(MoO_4_)_3_-active sites [62]. The regenerated Fe_2_(MoO_4_)_3_ FTIR spectrum, hereby denoted as (Fe_2_(MoO_4_)_3_–MB-Reg), obtained upon thermal treatment was very much comparable with that of fresh Fe_2_(MoO_4_)_3_, indicating the complete combustion of the attached MB on the surface. In addition, the obtained spectrum confirmed the purity of the regenerated material and the efficiency of the reused adsorbent. In the same sense, the X-ray diffraction (XRD) pattern of iron molybdate was taken before and after the regeneration, showing the same results.

### 2.5. MB Removal Mechanism

**Figure 12:**
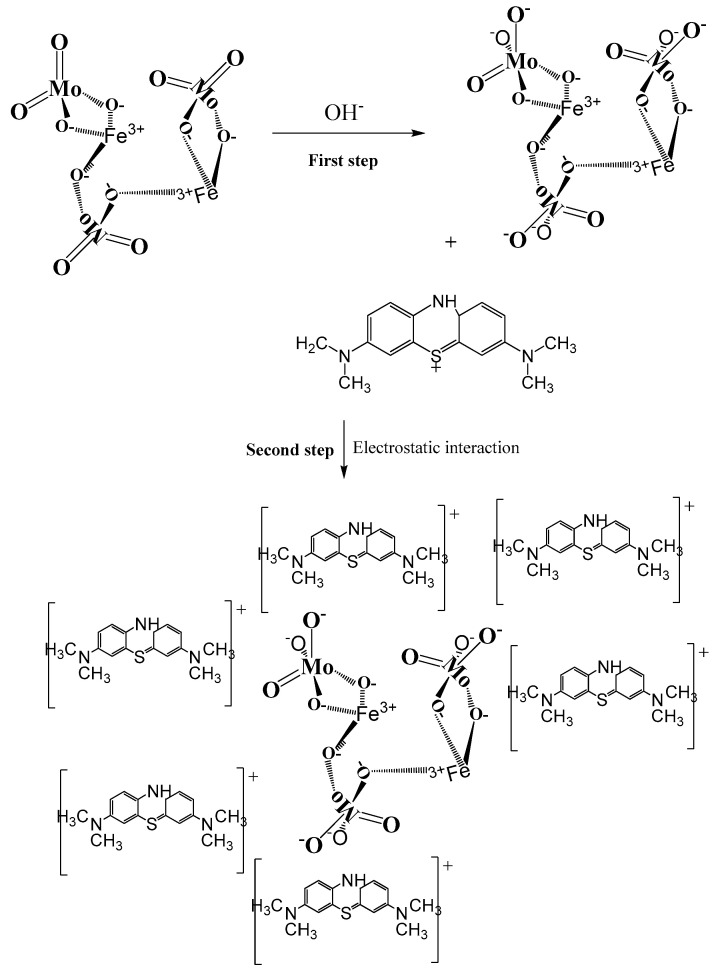
Schematic mechanism of MB dye removal using the iron molybdate nanosorbent.

As discussed earlier, MB removal by Fe_2_(MoO_4_)_3_ nanoparticles was found to proceed via an adsorption mechanism. In this respect, FTIR spectroscopic data depicted that no chemical decomposition of MB took place during its removal upon adsorption of the dye’s cations, and there was no evidence of any intermediate compounds. Moreover, the removal effectiveness of MB using Fe_2_(MoO_4_)_3_ nanoparticles increased with increasing pH values up to 11, which were attributed to the alkaline media. From these findings, we proposed the removal mechanism given in Figure 12. During the first step, MB (pKa = 3.8) maintained its positive charge at pH 11 [40]. In the same conditions, the iron molybdate (Fe_2_(MoO_5_^2−^)_3_) ion was produced, without intermediate compounds, by the reaction of Fe_2_(MoO_4_)_3_ with the hydroxyl groups (OH^−^) present in solution [63]. Hence, the electrostatic interactions were governing the adsorptive process. These strong interactions were notable between the negatively charged iron molybdate (Fe_2_(MoO_5_^2−^)_3_) surface and the positively charged MB cations [45].

**Figure 13:**
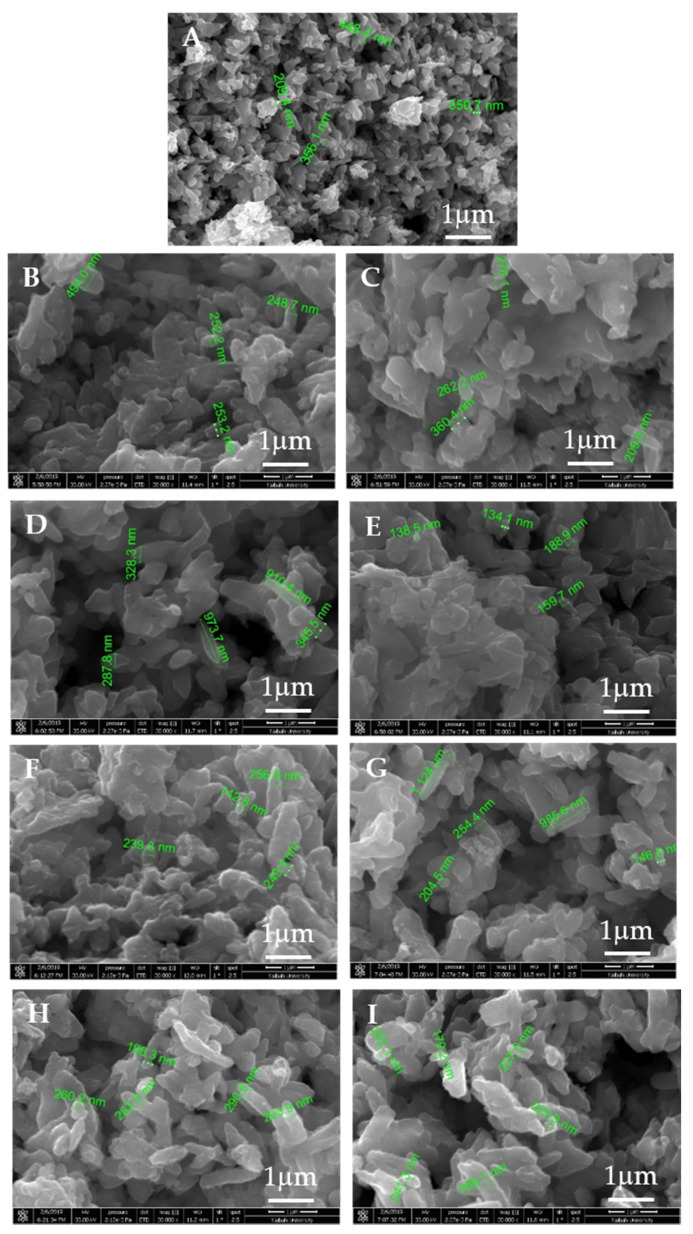
SEM micrographs of iron molybdate (Fe_2_(MoO_4_)_3_): (**A**) the starting pure iron molybdate, (**B**) after MB dye removal, (**C**) after the first regeneration, (**D**) after the first removal cycle of MB dye, (**E**) after the second regeneration process, (**F**) after the second removal cycle of MB dye, (**G**) after the third regeneration process, (**H**) after the third removal cycle of MB dye and (**I**) the morphology of Fe_2_(MoO_4_)_3_ after the final regeneration process.

To gain insights, at each adsorption step, into the morphological evolution of the Fe_2_(MoO_4_)_3_ nanosorbent, SEM micrograph images were taken, as shown in Figure 13, which provided some indication of how the starting pure iron molybdate (Fe_2_(MoO_4_)_3_) particles formed aggregates and showed good porosity, which could permit better adsorption of the dye (Figure 13A). However, the micrographs in Figure 13B,D,F,H indicated a less porous powder after the adsorption tests, i.e., the MB molecules filled the pores existing in the starting samples. Figure 13C,E,G,I showed that the sample morphology was not changed after regeneration and the first, second and third reuse. For all three cases, these less-agglomerated particles manifested as extremely porous powder. Generally, the morphology of Fe_2_(MoO_4_)_3_ was not considerably altered, even after the second or third reuse, as shown in Figure 13G,I.

## 3. Experimental

### 3.1. Iron Molybdate Nanosorbent Preparation

All chemicals were purchased from Sigma-Aldrich (St. Louis, MO, USA) and used as received without any changes, except for the methylene blue (MB) dye, which was supplied by Panreac, Barcelona, Spain.

Iron molybdate nanosorbent (Fe_2_(MoO_4_)_3_) was produced by iron molybdenum complex thermal breakdown, in the solid state, by reacting iron nitrate (Fe(NO_3_)_3_·9H_2_O), oxalic acid (H_2_C_2_O_4_·2H_2_O) and ammonium molybdate ((NH_4_)_6_Mo_7_O_24_·4H_2_O), as reported previously in the literature [22]. Iron nitrate (Fe(NO_3_)_3_·9H_2_O), oxalic acid (H_2_C_2_O_4_·2H_2_O) and ammonium molybdate ((NH_4_)_6_Mo_7_O_24_·4H_2_O) were mixed together in a molar ratio of 2:10:0.43. The obtained homogeneously powdered mixture was heated at 160 °C on a hot plate. The iron molybdenum complex obtained was then decomposed in a tubular furnace (with both ends open) at 500 °C under static air for two hours.

### 3.2. Adsorption Investigations

Methylene blue (MB) dye removal was investigated in batch equilibrium experiments [38]. The MB solution pH was controlled by the addition of either 0.01-N HCl or 0.01-N NaOH solutions. The removal of MB by Fe_2_(MoO_4_)_3_ was conducted with continual stirring of a specific quantity of the nanosorbent in MB solution (V = 100 mL) with known concentrations at various temperatures (T = 25 °C, 50 °C and 70 °C) and for different contact times (10 min, 30 min, 60 min, 90 min and 120 min). Next, a 0.22-µm (Whatman) syringe filter was employed to filter the solution, which was then investigated by UV-visible spectrometry at λ_max_ = 665 nm (Thermo Fisher Scientific, Madison, WI, USA). The percentage removed (%) and the amount of MB removed at equilibrium (q_e_ (mg/g)) were determined using the following equations:

(13) $$\mathrm{Removal}\;\%=\frac{C_{0}-C_{e}}{C_{0}}\times 100$$

(14) $$q_{e}=\frac{\left(C_{0}-C_{e}\right)}{M}\times V$$

where C_0_ and C_e_ (ppm) are the initial and equilibrium concentrations of MB, respectively, M (g) is the added mass of Fe_2_(MoO_4_)_3_ and V (L) is the volume of solution used. The results were reported in triplicate.

### 3.3. Method for Adsorbent Regeneration

For the experiments of adsorbent regeneration, an extended equilibrium time of 1 h was allocated for the removal with a 60-ppm solution that was used. The fresh Fe_2_(MoO_4_)_3_ used was calcined for 1 h at 400 °C under atmospheric air after being dried at 100 °C upon filtration. The calcined Fe_2_(MoO_4_)_3_ was tested for recycling purposes with the same conditions as that of freshly used Fe_2_(MoO_4_)_3_. The whole regeneration cycle was repeated thrice under the same conditions. The removed percentage (%) and the MB dye amount removed at equilibrium (q_e_ (mg/g)) were determined using Equations (13) and (14).

### 3.4. Characterization

**Figure 14:**
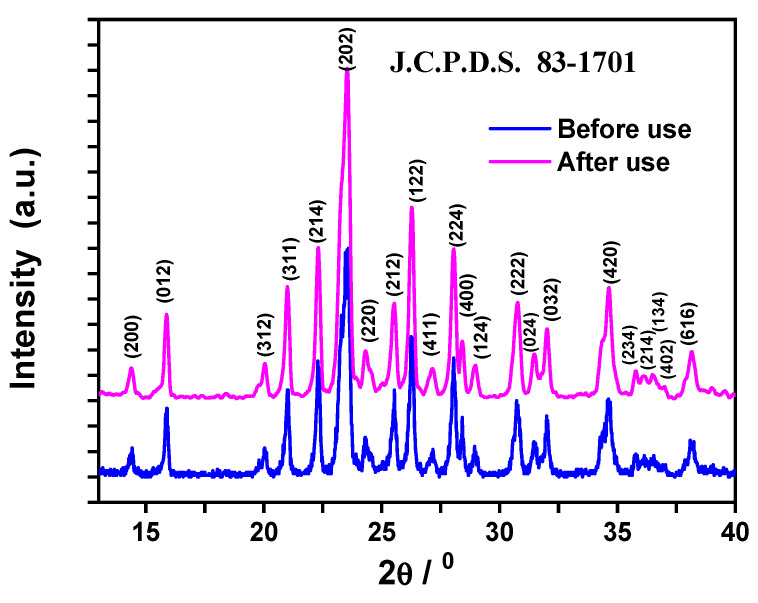
X-ray diffraction pattern of the synthetized Fe_2_(MoO_4_)_3_ nanoparticle powder before and after its use for the removal of MB dye. The Joint Committee on Powder Diffraction Standards (J.C.P.D.S) index file is 83-1701.

Analysis of XRD (λ_Cu-Kα_ = 1.5406 Å and Ni filter on a Shimadzu X-ray diffractometer 6000, Tokyo, Japan) was carried out for the identification of the synthetized Fe_2_(MoO_4_)_3_ nanosorbent material before and after its use for the removal of MB dye, as presented in Figure 14. The Scherrer equation was used to estimate the particle size from the XRD pattern of the as-prepared nanoparticles as follows: D_XRD_ = 0.9λ/(βcos θ), where D_XRD_ is the average particle diameter, λ is the Cu kα wavelength, β is the full-width at half-maximum (FWHM) of the diffraction peak and θ is the diffraction angle. The first two peaks were used to calculate the crystallite size D_XRD_, and the crystallite size was found to be 45 nm in both cases.

The nitrogen adsorption isotherm was employed for the determination of the specific surface area [22] with a value close 8.03 m^2^/g.

The presence of MB dye molecules on the Fe_2_(MoO_4_)_3_ nanoparticles was confirmed by FTIR spectroscopy, using the KBr pellet technique in the range of 400 to 4000 cm^−1^, on a Shimadzu apparatus (IR Affinity-1S, Shimadzu, Tokyo, Japan).

A Quanta FEG 250 scanning electron microscope (SEM; Thermo Fisher Scientific, Hillsboro, OR, USA) was used to study the surface morphology and the particle sizes of the synthesized materials.

The concentration at equilibrium of the MB dye was determined using a UV-visible spectrophotometer (Thermo Scientific Genesys 10S, Madison, WI, USA).

## 4. Conclusions

Fe_2_(MoO_4_)_3_ nanosorbent was synthesized and utilized as an MB removal agent in aqueous solutions. A strongly pH-dependent removal was observed, with an achieved removal efficiency of 99% after only 120 min of contact time at pH 11, using an initial dye concentration of 50 to 60 ppm. Further kinetic investigations revealed that MB removal followed a pseudo-second-order model, while the thermodynamic study showed that the Langmuir isotherm was the best fitted model to the experimental adsorption data. Interestingly, Langmuir model-based calculations showed that the removal capacity attained a maximum of 6173 mg/g. Efficient regeneration was possible upon calcination at 400 °C, and afterwards, the nanosorbent was ready for further reuse. The Fe_2_(MoO_4_)_3_ removal efficiency for MB was higher even after three cycles of reuse. The data showed that Fe_2_(MoO_4_)_3_ was indeed as an effective nanosorbent, endowed with excellent removal performance for the studied MB dye, and that it was not altered after several recycling tests.

## Figures and Tables

**Table 1:** Methylene blue (MB) removal by Fe_2_(MoO_4_)_3_ thermodynamic parameters.

Adsorbent	Adsorbate	∆H° (KJ·mol^−1^)	∆S° (KJ·mol^−1^·K)	∆G° (KJ·mol^−1^)		
Fe_2_(MoO_4_)_3_	MB	83.79	0.291	296K	323K	343K
				−2.904	−8.934	−16.402

**Table 2:** Kinetic model equations.

Model	Equation	Parameters
Pseudo-first-order (PFD) [50]	$\ln\left(q_{e}-q_{t}\right)={\ln\;q}_{e}+K_{1}t$ (4)	q_t_: the removal capacity at time t (mg/g) q_e_: the removal capacity at equilibrium (mg/g) K_1_: the rate constant of pseudo-first-order adsorption (1/min)
Pseudo-second-order (PSD) [50]	$\frac{t}{q_{t}}=\frac{1}{K_{2}q_{e}^{2}}+\frac{t}{q_{e}}$ (5)	q_t_: the removal capacity at time t (mg/g) q_e_: the removal capacity at equilibrium (mg/g) K_2_: the pseudo-second-order rate constant (g. mg^−1^.min^−1^)
Intraparticle diffusion (IPD) [51]	$q_{t}=K_{I}t^{0.5}+l$ (6)	I (mg/g) and K_I_ (mg/(g.min^0.5^)) are the intraparticle diffusion constants q_t_: the removal capacity (mg/g) at time t t: the contact time (min)

**Table 3:** MB removal by Fe_2_(MoO_4_)_3_ kinetic parameters.

Dye (C_i_ mg/L)	Pseudo-First-Order				Pseudo-Second-Order			Intraparticle Diffusion Model		
	q_exp_ (mg/g)	q_e_ (mg/g)	k_1_ (1/min)	R_1_^2^	q_e_ (mg/g)	k_2_ (g/mg min)	R_2_^2^	I (mg/g)	k_i_ (mg/g min^0.5^)	R_3_^2^
50	4999	378	0.020	0.956	4981	0.00021	1.000	4596	36	0.904
60	5967	496	0.014	0.814	5871	0.00016	0.999	5444	37	0.782
65	5979	565	0.019	0.752	5922	0.00016	1.000	5135	85	0.748
70	6179	57	0.021	0.993	6176	0.00154	1.000	6117	6	0.993

**Table 4:** Adsorption isotherm models for MB dye removal using Fe_2_(MoO_4_)_3_.

Model	Equation	Parameters
Freundlich [52]	${\ln\;q}_{e}={\ln\;q}_{F}+\frac{1}{n}{\ln\;C}_{e}$ (7)	q_F_: Freundlich constant (mg^(1−1/n)^L^1/n^g^−1^) n: heterogeneity factor (g/L) q_e_: amount of MB dye adsorbed by α-Fe_2_(MoO_4_)_3_ at equilibrium (mg/g) C_e_: MB concentration at equilibrium (ppm)
Langmuir [52]	$\frac{C_{e}}{q_{e}}=\frac{1}{q_{m}K_{L}}+\frac{C_{e}}{q_{m}}$ (8)	q_e_: amount of MB dye adsorbed by α-Fe_2_(MoO_4_)_3_ at equilibrium (mg/g) C_e_: MB concentration at equilibrium (ppm) q_m_: maximum amount of MB dye removed by Fe_2_(MoO_4_)_3_ (mg/g) K_L_: Langmuir adsorption constant (L/mg)
	$R_{L}=\frac{1}{1+K_{L}C_{i}}$ (9)	C_i_: initial concentration of MB K_L_: Langmuir constant R_L_: values specify that the removal of MB dye could be linear (R_L_ = 1), irreversible (R_L_ = 0), favorable (0 < R_L_ < 1) or unfavorable (R_L_ > 1)
Dubinin-Radushkevich (D-R) [53]	${\ln\;q}_{e}={\ln\;q}_{m}-K\varepsilon^{2}$ (10) $\varepsilon =RT\ln\left(1+\frac{1}{C_{e}}\right)$ (11)	K: sorption energy constant (mol^2^/kJ^2^) ε: Polanyi potential T: temperature (K) R: universal gas constant (8.314 J.mol^−1^ K^−1^) q_m_: theoretical saturation capacity C_e_: MB concentration at equilibrium (ppm)
Temkin [54]	$q_{e}=B_{T}{\ln\;A}_{T}+B_{T}{\ln\;C}_{e}$ (12)	b_T_: Temkin constant related to heat of sorption (J/mol), B_T_ = R_T_/b_T_ R: gas constant (8.314 J/mol K) A_T_: Temkin isotherm constant (L/g) T: absolute temperature (K)

**Table 5:** MB removal by Fe_2_(MoO_4_)_3_ isotherm parameters.

Langmuir				Freundlich			Temkin			Dubinin-Radushkevich		
q_m_ (mg/g)	K_L_ (L/mg)	R^2^	Range R_L_	q_F_ (mg^(1−1/n)^L^1/n^g^−1^)	1/n	R^2^	A_T_ (L/g)	B_T_	R^2^	q_m_ (mg/g)	R^2^	E (Kj/mol)
6173	5	0.999	0.0024–0.0038	5825	0.02	0.866	5E18	135	0.870	6063	0.971	944

**Table 6:** The maximum removed amount (q_m_) of MB dye reported in the literature.

Nanosorbent	q_m_ (mg/g)	Reference
Magnetic β-cyclodextrin-chitosan nanoparticles	2783.30	[55]
Fe_2_O_3_	1124.70	[56]
Zinc molybdate nanoparticles	217.86	[23]
CoO	5501.93	[56]
Molybdenum trioxide nanorods and stacked nanoplates	152.00	[45]
Iron molybdate (Fe_2_(MoO_4_)_3_)	6173.00	This work
